# Recent Advances of Acute Kidney Injury in Hematopoietic Cell Transplantation

**DOI:** 10.3389/fimmu.2021.779881

**Published:** 2022-01-04

**Authors:** Masahiro Miyata, Kazunobu Ichikawa, Eri Matsuki, Masafumi Watanabe, Daniel Peltier, Tomomi Toubai

**Affiliations:** ^1^ Department of Cardiology, Pulmonology, and Nephrology, Faculty of Medicine, Yamagata University, Yamagata, Japan; ^2^ Department of Pediatric Hematology/Oncology, University Michigan Medical School, Ann Arbor, MI, United States; ^3^ Department of Internal Medicine III, Division of Hematology and Cell Therapy, Faculty of Medicine, Yamagata University, Yamagata, Japan

**Keywords:** acute kidney injury, allogeneic hematologic stem cell transplantation, GvHD, experimental BMT, cytokine, calcinurin inhibitors, thrombotic microagiopathy

## Abstract

Acute kidney injury (AKI) is a common complication of allogeneic hematopoietic cell transplantation (allo-HCT) and is associated with non-relapse mortality (NRM) and quality of life (QOL). Multiple factors may contribute to AKI during allo-HCT and are often present at the same time making it difficult to determine the cause of AKI in each patient. Nephrotoxic drugs, infections, thrombotic microangiopathy (TMA), and sinusoidal obstruction syndrome (SOS) are well described causes of AKI during allo-HCT. Acute graft-versus-host disease (aGVHD) is a major complication of allo-HCT that mainly targets the intestines, liver, and skin. However, recent studies suggest aGVHD may also attack the kidney and contribute to AKI following allo-HCT. For example, severe aGVHD is associated with AKI, suggesting a link between the two. In addition, animal models have shown donor immune cell infiltration and increased expression of inflammatory cytokines in recipient kidneys after allo-HCT. Therefore, aGVHD may also target the kidney and contribute to AKI following allo-HCT. Herein, we describe the etiology, diagnosis, risk factors, pathophysiology, prevention, and treatment of renal injury after allo-HCT. In addition, we highlight emerging evidence that aGVHD may contribute to the development of AKI after allo-HCT.

## Introduction

Hematopoietic cell transplantation (HCT) is a curative therapy for hematologic malignancies and many non-malignant diseases (1). HCT is classified as either autologous (auto-HCT), when recipient hematopoietic stem cells are stored and then infused, or allogeneic (allo-HCT), when the infused hematopoietic stem cells are derived from a related or unrelated donor. Prior to transplantation, conditioning with chemotherapy and/or total body irradiation (TBI) is necessary to eradicate malignant residual tumors and inhibit rejection of donor hematopoietic cells. Myeloablative conditioning with high-dose cyclophosphamide (CY) and TBI or a combination of busulfan (BU) and CY are two common regimens. Non-myeloablative conditioning or reduced-intensity conditioning with less intense pretreatment is commonly utilized for elderly patients or those with comorbidities (2).

To prevent graft-versus-host disease (GVHD), immunosuppressive prophylaxis is necessary after transplantation (3). GVHD is caused by alloreactive donor T cells, attacking recipient tissues and is a major life-threatening complication of allo-HCT (4). Previously GVHD was classified into acute GVHD (aGVHD) if it developed within 100 days after transplantation or chronic GVHD (cGVHD) if it developed after 100 days. However, GVHD classification is now based on clinical and pathological characteristics (5).

The characteristic symptoms of aGVHD are rash, diarrhea, and jaundice (4). The pathophysiology causing these symptoms begins with tissue damage from conditioning regimens that results in the release of damage-associated molecular patterns (DAMPs). Injury to the intestinal mucosa and skin also causes a breakdown in barrier function. Barrier breakdown allows microbes to invade the body and release pathogen-associated molecular patterns (PAMPs). PAMPs and DAMPs are danger signals that activate the innate immune system to produce proinflammatory cytokines, such as tumor necrosis factor (TNF)-α and interleukin (IL)-1β, which amplify tissue damage and activate antigen-presenting cells (APCs). Activated host APCs then stimulate donor T cells, which in turn produce proinflammatory cytokines, such as interferon (IFN)-γ, that further activate the innate immune system. Finally, tissue damage caused by cytotoxic T lymphocytes and cytotoxic cytokines derived from activated T cells and innate immune cells, results in the development of clinically apparent aGVHD (3, 6).

The most common immunosuppressive regimen used to prevent GVHD after allo-HCT consists of a calcineurin inhibitor (tacrolimus or cyclosporine) in combination with a short-term course of methotrexate (MTX) (4). Systemic high-dose corticosteroids are the first-line treatment for patients who develop aGVHD (7).

The main organs affected by aGVHD are the skin, liver, and gastrointestinal (GI) tract, but a variety of other organs may also be affected (8). Classically, the kidney is not recognized as a main target organ of aGVHD and no renal aGVHD diagnostic criteria have been established (8). However, various factors related to conditioning and GVHD prophylaxis are known to cause renal injury after allo-HCT. In patients with aGVHD and renal dysfunction, it is often difficult to identify the cause of renal dysfunction due to the frequent co-occurrence of multiple possible etiologies. Renal biopsy is the gold-standard examination for deconvoluting multiple possible etiologies of renal injury; however, invasive renal biopsies are rarely safe during the acute phase of GVHD. Recently, animal studies suggest that the kidney may be a target of aGVHD. Here, we describe the pathophysiology and management of acute kidney injury (AKI) after allo-HCT and highlight the emerging association between AKI and aGVHD.

## Criteria for Acute Kidney Injury

Although AKI is a common disease, there have been no internationally standardized criteria (9). In 2004, the Acute Dialysis Quality Initiative (ADQI) published the Risk, Injury, Failure, Loss, and End-stage renal disease (RIFLE) criteria (10). The ADQI defined acute renal failure (ARF) as elevated serum creatinine (sCr), decreased glomerular filtration rate (GFR), and decreased urine output. The AQDI also classified the severity of ARF based on the degree to which these parameters were altered (10). Later, the Acute Kidney Injury Network (AKIN) proposed the concept of acute kidney injury (AKI) in order to include early renal injury. The AKIN criteria, published in 2007, modified the RIEFLE criteria by including mild elevations of sCr (11). In 2012, the Kidney Disease Improving Global Outcomes (KDIGO) criteria were proposed, which integrated the RIEFLE and AKIN criteria. The KDIGO criteria for AKI include anyone of the following: 1) an increase in sCr by≧0.3 mg/dl within 48 hours, 2) an increase in sCr to ≧1.5 times baseline within the preceding 7 days, and 3) a urine volume <0.5 ml/kg/h for 6 hours. The severity of AKI is classified by the KDIGO as Stage 1 to 3 based on sCr or urine output (12). The details of each criterion are shown in **Table 1**. Importantly, the latest KDIGO criteria are as or more predictive of life expectancy than either the RIEFLE or AKIN criteria (13–15). The KDIGO criteria are frequently used in recent studies to measure the incidence of AKI after HCT (16–19).

**Table 1:** Classification of AKI severity.

	Serum creatinine	Urine output
**RIFLE**		
Risk	Increase sCr ×1.5 or GFR decrease > 25%	<0.5 ml/kg/h for 6 hours
Injury	Increase sCr ×2 or GFR decrease > 50%	<0.5 ml/kg/h for 12 hours
Failure	Increase sCr ×3 or GFR decrease 75% or sCr > 4 mg/dl	<0.3 ml/kg/h for 24 hours or Anuria for 12 hours
Loss	Complete loss of kidney functions > 4 weeks	
ESKD	End Stage Kidney Disease >3 months	
**AKIN**		
Stage 1	Increase ≧0.3 mg/dl or 1.5-2 fold from baseline	<0.5 ml/kg/h for 6 hours
Stage 2	2-3 fold from baseline	<0.5 ml/kg/h for 12 hours
Stage 3	>3 fold from baseline or sCr ≧ 4.0 mg/dl with an acute increase of at least 0.5 mg/dl	<0.3 ml/kg/h for 24 hours or Anuria for 12 hours
**KDIGO**		
Stage 1	1.5–1.9 times or ≧0.3 mg/dl increase	<0.5 ml/kg/h for 6 hours
Stage 2	2.0–2.9 times	<0.5 ml/kg/h for 12 hours
Stage 3	3.0 times or Increase to ≧4.0 mg/dl or initiation of renal replacement therapy or, in patients <18 years, decrease in eGFR to <35 ml/min/1.73 m^2^	<0.3 ml/kg/h for 24 hours or Anuria for 12 hours

RIFLE, Risk, Injury, Failure, Loss, and End-stage renal disease; AKIN, Acute Kidney Injury Network; KDIGO, Kidney Disease Improving Global Outcomes; sCr, serum creatinine; GFR, glomerular filtration rate.

## Kidney Disease After HCT

According to a recently published meta-analysis of reports from 1995-2019, the incidence of AKI after HCT was 55.1%, with Stage 3, the most severe form, occurring in 8.3% of patients (20).

Factors known to contribute to the risk of AKI after HCT include pre-treatment factors such as being female (21), age 55 years or older (22), and underlying conditions such as diabetes (23), hypertension (21), and chronic kidney disease (CKD) (24). Risk factors for AKI associated with HCT include TBI conditioning (22), use of a calcineurin inhibitors (CNIs) for GVHD prevention (23, 25, 26), and use of MTX for GVHD prophylaxis (22, 23). Post-transplant stay in an intensive care unit (21) and the need for mechanical ventilation (27) are also risk factors. Several post-HCT complications also increase the risk for AKI including hepatic sinusoidal obstruction syndrome (SOS) (28), sepsis (28, 29), and cytomegalovirus infection (22). AKI risk can be further increased by agents used to treat post-HCT complications including amphotericin B (30), acyclovir (31), aminoglycosides (32), and the concomitant use of multiple nephrotoxic drugs (33).

AKI is more common in the early phase of HCT due to the risk of conditioning toxicity, sepsis, SOS, and drug-induced renal injury that are more common early post-HCT (23, 34). For these reasons, clinical studies typically assess post-HCT AKI at 100 days post-transplantation (35).

The incidence of AKI varies according to the type of HCT. In auto -HCT recipients, graft failure is less common, and CNIs are not required because there is no risk for GVHD. Less antibiotics are administrated to auto-HCT recipients than to allo-HCT recipients because duration of neutrophilia is shorter. Therefore, the incidence of AKI is less in auto-HCT versus allo-HCT recipients (36–39). AKI incidence is lower following nonmyeloablative compared to myeloablative conditioning due to decreased rates of infection, SOS, and organ failure (35, 40). Overall, the greatest risk of AKI is with myeloablative allo-HCT (21-73%), followed by nonmyeloablative allo-HCT (29-56%), and then autologous transplantation (10.4-19%) (36, 41, 42). Reports vary on whether the incidence of AKI is higher with cord blood or HLA mismatched donor transplantation (28, 43–47).

Whether the indication for HCT is for a malignant or nonmalignant disorders does not significantly affect the incidence of AKI (33, 48, 49). However, malignancies are a risk factor for post-transplant CKD (50), and these patients should be monitored carefully for changes in renal function. Multiple myeloma (MM) and immunoglobulin light-chain (AL) amyloidosis, which themselves cause AKI (see the section of “*Etiologies of AKI after HCT*”), have been reported to cause relatively little post-transplant AKI. However, patients with these disorders are typically treated with auto-HCT, which is less nephrotoxic than allo-HCT (19, 24).

AKI after allo-HCT is associated with all-cause (19, 20, 22, 28, 33, 39, 51) and non-relapse mortality (17, 23, 33), and the earlier the onset of AKI, the higher the mortality (52). The severity of AKI decreases overall survival, and mortality worsens to 55-100% with renal failure requiring dialysis (17, 22, 36, 37, 53, 54). AKI after allo-HCT is also a risk factor for CKD (33, 50). Representative studies that focus on the association of AKI with transplant outcomes are shown in **Table 2**. In pediatric HCT recipient, the incidence of AKI is similar to adults (44, 48, 49, 56–58), AKI worsens mortality after HCT (49, 57), and the 1-year survival rate is less than 10% in patients with renal failure requiring renal replacement therapy (56). Fortunately, HCT-related AKI has decreased in recent years due to the increased use of less toxic conditioning regimens, decreased rates of SOS, modified infection prophylaxis, less amphotericin B use, and declining rates of severe aGVHD (59, 60).

**Table 2:** Recent studies on the association of AKI with transplant outcomes.

Study	Year	Type of transplantation	AKI definition	Incidence of AKI	Follow up	Overall mortality (non-AKI vs AKI)	Non-relapse mortality (non-AKI vs AKI)
Mori et al. (54)	2012	allo-HCT	AKIN	62.2%	5 years	25% vs 45%, HR for death; >Stage 3 vs no AKI or stage 1-2; 5.49 (p <0.001)	NA
Sehgal et al. (37)	2017	allo-HCT, auto-HCT(16.9%)	RIFLE	75.4%	3 months	non-AKI 17.6%, risk 40%, injury 36.4%, failure 80% (p=0.027)	NA
Piñana et al. (17)	2017	allo-HCT(RIC)	KDIGO	44%	25 months	non-AKI 22%, grade 1 32%, grade 2 50%, grade 3 70% (p<0.0001)	16% vs 33% (p=0.005)
Liu et al. (55)	2018	haplo-HCT	sCr>1.5-fold rise	43%	2 years	non-AKI 21.1% vs grade 3(sCr>3-fold) 55.4% (p<0.001)	PFS; non-AKI 72.2% vs severe AKI 45.7 (p<0.001)
Khalil et al. (38)	2019	allo-PBSCT, aotu-PBSCT(38.6%)	RIFLE	31.6%	3 months	17% vs 42% survival time; non-AKI 130 vs injury or failure 38 months (p=0.001)	NA
Mima et al. (18)	2019	allo-HCT, auto-HCT(14.8%)	KDIGO	15.7%	100 days	20.2% vs 29.4% (p=0.409)	NA
Andronesi et al. (19)	2019	auto-HCT	KDIGO	10.3%	90 days	0.6% vs 5.3% (p=0.01)	NA
Sakaguchi et al. (33)	2020	allo-HCT	KDIGO	64.9%	5 years	42.7% vs 76.2% (p<0.001)	13.3% vs 59.8% (p<0.001)
Gutiérrez-García et al. (22)	2020	allo-HCT	KDIGO	58%	5 years	AKI 0-1-2, 55% vs AKI-3, 70% (p=0.008)	TRM; AKI 0-1-2, 31% vs AKI-3, 51% (p<0.0001)
Bhasin et al. (39)	2021	auto-HCT(56.1%), allo-HCT	increase in sCr > 0.3 mg/dL	23%	100 days	1.4% vs 15.6% (p<0.001)	NA

AKI, acute kidney injury; HCT, hematopoietic cell transplantation; allo, allogeneic; auto, autologous; RIC, reduced-intensity conditioning; PBSCT, peripheral blood stem cell transplantation; AKIN, Acute Kidney Injury Network; RIFLE, Risk, Injury, Failure, Loss, and End-stage renal disease; KDIGO, Kidney Disease Improving Global Outcomes; sCr, serum creatinine; HR, hazard ratio; PFS, progression free survival; TRM, transplant-related mortality. NA, not applicable.

## Etiologies of AKI After HCT

**Figure 1:**
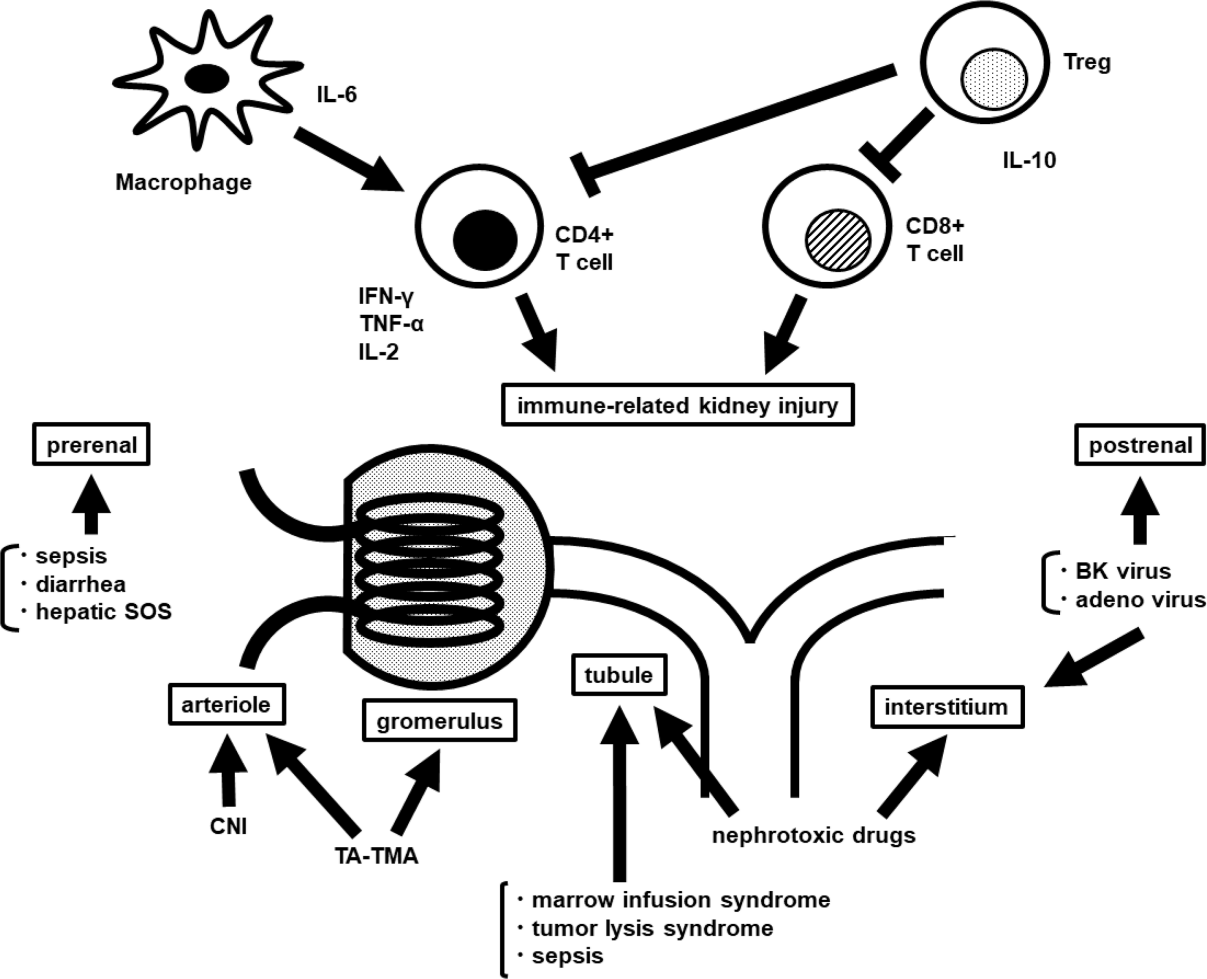
Overview of the pathophysiology of AKI after HCT. AKI, acute kidney injury; HCT, hematopoietic cell transplantation; IL, interleukin; IFN, interferon; TNF, tumor necrosis factor; Treg, regulatory T cell; SOS, sinusoidal obstruction syndrome; CNI, calcineurin inhibitor; TA-TMA, transplantation associated-thrombotic microangiopathy.

There are various causes of AKI after HCT. An overview is shown in **Figure 1**.

### Nephrotoxic Drugs

Most of the renal injury after HCT is thought to be caused by nephrotoxic drugs, particularly CNIs given for GVHD prophylaxis (34). CNIs can cause both AKI and CKD (61); however, CNIs serum concentration does not always correlate with the severity of AKI (26). CNIs cause AKI through a variety of mechanisms. One way is by inhibiting the production of vasodilators and increasing the production of vasoconstrictors, resulting in the contraction of afferent and efferent arterioles. They also cause vacuolation and dysfunction of renal tubules (61), and they increase the levels of oxidative stress that damages the renal endothelium and contributes to the development of thrombotic microangiopathy (TMA) (62). Consistent with the vasoconstrictive effect of CNIs on afferent and efferent arterioles, inhibition of the renin-angiotensin-aldosterone system may be useful for preventing CNI nephrotoxicity (60).

MTX can also be nephrotoxic. The mechanism is thought to relate to direct tubular injury and/or its precipitation in the renal tubules. The risk for MTX-induced nephrotoxicity is increased by high dose intravenous administration, dehydration, and aciduria (63).

Chemotherapeutic agents used in conditioning (cytarabine and fludarabine) can be nephrotoxic and primarily cause acute tubular injury. Vomiting and diarrhea, which are common adverse events of chemotherapy, cause pre-renal AKI due to dehydration (35). CY and BU cause post-renal AKI due to hemorrhagic cystitis (35, 64).

Many antimicrobial agents may induce direct renal injury or acute interstitial nephritis due to allergic reactions leading to AKI (60). For example, aminoglycosides can cause Fanconi syndrome and Bartter-like syndrome (32). The antifungal agent, amphotericin B causes AKI in a dose-dependent manner *via* renal vasoconstriction and direct tubular injury (65). Fortunately, liposomal amphotericin B, which is now more commonly used, is far less nephrotoxic (66). Finally, acyclovir, an antiviral agent, has been associated with crystal-induced tubular injury and obstruction (60, 67).

### Hematological Disease-Associated AKI

Hematologic diseases themselves can cause renal injury. For example, AKI occurs in 20-50% of patients with multiple myeloma (68). The most common cause of AKI in patients with multiple myeloma is cast nephropathy, in which large amounts of light chains bind to Tamm-Horsfall protein in the tubules and form insoluble casts, resulting in tubular obstruction and injury. Other causes include light chain deposition disease, AL amyloidosis, and hypercalcemia (69). Direct invasion of the kidney by lymphoma and leukemia can also cause AKI (70).

### Complications of HCT

Recipients are immunocompromised and prone to sepsis after HCT (60). Gram-negative bacteria is more common in cord blood transplantation than in bone marrow transplantation (71), likely due to a longer period of neutropenia. Sepsis causes systemic vasodilatation, hypotension, and cytokine-induced endothelial damage, leading to AKI (72). As mentioned above, some of the antimicrobial agents used in the treatment of sepsis are nephrotoxic.

Adenovirus and BK virus infections are also common opportunistic infections following allo-HCT and often lead to AKI (35, 60). Adenoviruses may cause hemorrhagic cystitis and, rarely, necrotizing tubulointerstitial nephritis (73). Adenovirus infections are more common in transplants from unrelated donors and in pediatric patients. Severe adenovirus infection can cause hepatitis, pneumonitis, and encephalitis, and multi-organ failure (74). Reactivation of BK virus may lead to hemorrhagic cystitis, ureteral stricture, and tubulointerstitial nephritis (75). Acute GVHD, allo-HCT, and BK viremia are still associated with increased risk for hemorrhagic cystitis (76, 77).

Nephrotic syndrome, while rare (0.4-6.0%), may also develop following allo-HCT (34). Membranous glomerulonephritis (MGN) and minimal change disease (MCD) account for about two-thirds and one-quarter of nephrotic syndrome cases following allo-HCT, respectively (78). Intriguingly, the onset of nephrotic syndrome following allo-HCT has been associated with recent reduction in the dose of immunosuppressive drugs (78) and the onset of GVHD (79). Most cases occur more than 6 months after transplantation and are considered a rare manifestation of cGVHD (34, 60). However, *de novo* nephrotic syndrome without GVHD also occurs (80).

Marrow infusion syndrome is caused by hemolysis of erythrocytes that release hemoproteins into the recipient. These hemoproteins cause symptoms of hemolysis, such as fever and vomiting. They can also cause acute tubular injury by forming casts in the tubules. Hemolysis resulting in marrow infusion syndrome often occurs during the preservation of stem cells or upon infusion of grafts containing the cryoprotectant dimethyl sulfoxide (DMSO), which can cause hemolysis in recipients of DMSO-containing grafts. Marrow infusion syndrome is mitigated and treated by intravenous hydration and by rinsing or red blood cell-depleting the graft (81).

Tumor lysis syndrome (TLS) occurs when a large number of tumor cells lyse and release toxic cellular contents. It is characterized by hyperkalemia, hypocalcemia, hyperphosphatemia, hyperuricemia, and crystal-induced kidney injury (82). While more common during induction chemotherapy for leukemia, TLS is relatively rare following HCT because most patients come to transplant following multiple treatment courses that dramatically reduce tumor burden (82).

Hepatic sinusoidal obstruction syndrome (SOS) is characterized by painful hepatomegaly, jaundice, and weight gain due to fluid retention (83). AKI often co-occurs with SOS (84, 85), which is more frequently seen after allo- than auto-HCT (81), and severe SOS may lead to multiple organ failure (86). SOS develops following sinusoidal endothelial damage from conditioning therapy, resulting in hepatic portal hypertension, ascites, and increased abdominal pressure. While the exact cause of renal injury in SOS is uncertain and likely multifactorial, decreased renal blood flow due to elevated abdominal pressure likely contributes to tubular injury, which further exacerbates fluid retention and multiorgan failure (86, 87).

### Thrombotic Microangiopathy

Transplantation-associated-thrombotic microangiopathy (TA-TMA) is another complication of HCT associated with a substantial risk of mortality (88). It typically develops subacute or chronically (89), and can also lead to AKI (60). Vascular endothelial damage associated with transplantation results in thrombus formation and fibrin deposition in the capillaries and small arteries, microangiopathic hemolytic anemia, and consumptive thrombocytopenia (88). TBI, high-dose BU, CNIs, aGVHD Grade II-IV, infections (BK virus, cytomegalovirus, parvovirus B19, aspergillus species, adenovirus), peripheral blood stem cell transplantation, and use of unrelated donors are all risk factors for TA-TMA (62).

Diagnostic criteria for TA-TMA have been developed by the Blood and Marrow Transplant Clinical Trial Network (BMT-CTN) (90) and the European Group for Blood and Marrow Transplantation (EBMT) (91). Both sets of criteria require the presence of schistocytes and elevated lactate dehydrogenase (LDH). The BMT-CTN criteria also requires worsening renal function (90).

The Kidney is the most vulnerable organ to TA-TMA (62, 89). Renal TA-TMA presents as both AKI and CKD (62, 89) and is often accompanied by hypertension, proteinuria, and a decreased GFR (89). The histopathology of renal TA-TMA is characterized by fibrin deposition in the glomeruli, narrowing of the capillary lumen, presence of fragmented red blood cells, basement membrane duplication, mesangiolysis, and edema of the endothelium (89, 92).

Although endothelial damage plays a major role in the pathogenesis of TA-TMA (62), it is unclear whether it is a direct complication of transplantation or a manifestation of GVHD, infection, or drug toxicity (60). Factors known to cause endothelial damage include CNIs, mammalian target of rapamycin (mTOR) inhibitors, chemotherapy, and TBI (35). Recent studies have also suggested the involvement of complement activation (62, 88).

Several clinical studies have shown an association between TA-TMA and aGVHD (93, 94), but these were retrospective studies confounded by the use of CNIs (88). Nevertheless, clinical studies have suggested that vascular endothelial cells are targeted by donor T cells (95), and some studies suggest that TA-TMA may be caused by GVHD of the vascular endothelium (89, 96, 97).

### Kidney Disease Associated With GVHD

Renal injury after allo-HCT is generally attributed to the etiologies describes above. However, aGVHD is a risk factor for AKI (23, 28, 53, 54, 98), and recent studies have suggested that the kidney may be a direct target of aGVHD (34). Traditionally, the kidney was not considered a target of aGVHD (35). However, diarrhea associated with severe GVHD can indirectly cause dehydration leading to AKI, and CNIs used for GVHD prophylaxis can also cause AKI. Hence, the association between kidney injury and aGVHD is controversial. In the following section, we review studies investigating the relationship between aGVHD and AKI.

#### Clinical Studies

Hingorani et al. (99) measured cytokines in the urine of patients who underwent allogeneic or autologous transplantation. Increased urine IL-6 and IL-15 levels after HCT were associated with an increased risk of developing proteinuria, and an increased urine MCP-1 level after HCT was associated with chronic kidney disease at 1 year. Thus, these data suggested kidney inflammation occurs after HCT.

Inflammatory cytokines are involved in the pathogenesis of GVHD, but they are not unique to GVHD and are elevated by other HCT-related complications and inflammatory disorders (100). In studies exploring GVHD-specific biomarkers, elafin was identified as a biomarker for cutaneous GVHD (101). Elafin is an elastase-specific protease inhibitor expressed mainly in epithelial tissues, is secreted in response to IL-1 and TNF-α, and has functions such as antibacterial activity, inflammatory cell recruitment, and dendritic cell activation (102, 103). In a study that measured urine elafin levels in patients after HCT (98), it was found that patients with AKI had higher urine elafin levels than those without AKI, and patients with albuminuria also had higher urine elafin levels than those without albuminuria. In addition, elafin was associated with increased risk of CKD and death. These data suggest that inflammation similar to cutaneous aGVHD may occur in the kidney.

Histological diagnosis of renal dysfunction shortly after HCT is rare, and pathological diagnostic criteria have not been established (35). Nonetheless, several renal histopathology studies using tissue obtained by biopsy or autopsy have been reported. For example, Girsberger et al. (104) reported that renal biopsy pathology was consistent with TA-TMA in 29%, CNI toxicity in 24%, and membranous glomerulonephritis in 18% of patients who presented with deterioration of kidney function or proteinuria after HCT (12 allo-HCT, 5 auto-HCT). In 137 autopsies (114 allo-HCT, 23 autologous HCT), the most common renal pathology was acute tubular damage (40%), followed by chronic vascular and interstitial change (11%), and TMA (10%). A small number of cases of membranous glomerulonephritis (1%) and acute interstitial nephritis (1%) were also observed. The median time from transplantation was 497 days for biopsies and 91 days for autopsies; therefore, cGVHD may have a greater association with kidney injury than aGVHD. Mii et al. (97) studied renal biopsy (two cases) and autopsy (5 cases) tissue from patients who developed renal TA-TMA. The median interval between HCT and renal biopsy or autopsy was 7 months. Five of the 7 patients underwent allo-HCT, all 7 patients underwent conditioning that included TBI, and all but one patient received a CNI for GVHD prophylaxis. In addition to TA-TMA changes, all patients had glomerulitis, tubulitis, and peritubular capillaritis with T cell infiltration. Based on these results, the authors concluded that the kidney is a potential target for GVHD.

#### Studies With Animal Models

Animal models have been important tools for studying the pathophysiology of HCT complications, most notably GVHD, and for developing new therapies to treat these complications (105). In addition to the above-described clinical studies, kidney injury associated with aGVHD has also been studied in animal models. These models revealed important insights into the relationship between kidney injury post allo-HCT and GVHD.

Two studies that measured renal function in mice after allo-HCT reported that sCr did not increase (106, 107). Because creatinine is a waste product of muscle metabolism (108), the lack of sCr elevation may have been due to loss of muscle mass after allo-HCT. By contrast, blood urea nitrogen (BUN), another marker of renal function, was elevated in a rat model (107).

In addition to markers of renal function, elevated markers of renal injury have also been observed in mouse models. These markers include urine protein, albumin (106), N-acetyl-beta-D-glucosaminidase (NAG) (106, 107), and neutrophil gelatinase-associated lipocalin (NGAL) (109), which mainly reflects tubular injury (110, 111). The expression of αKlotho, which is down-regulated in AKI and CKD (112), was also decreased in allo-HCT mice (109).

Higo et al. (107) evaluated renal lesions in a rat bone marrow transplantation (BMT) model. The kidneys in the allo-HCT group were infiltrated with donor leukocytes. Areas with mild inflammation were characterized by CD4+ T cell, CD8+ T cell, and CD68+ macrophage infiltration of the interstitium around small arteries. Whereas in lesions with moderate to severe inflammation, the cellular infiltrate extended into the interstitium surrounding the tubules. Peritubular capillaritis, tubulitis, acute glomerulitis, and endarteritis were also observed in lesions with moderate to severe inflammation. There was no renal deposition of immunoglobulin or complement. In a study using a mouse BMT model (106), similar results were reported. Specifically, allo-HCT recipient mice developed aGVHD within 4 weeks; renal tissue was infiltrated with CD4+ T cells, CD8+ T cells, FoxP3+ T cells, and macrophages; and endarteritis, interstitial nephritis, tubulitis, and glomerulitis were observed.

Ma et al. (113) observed the presence of renal TA-TMA in a TBI-conditioned murine BMT model. In the kidneys of allo-HCT recipients, in addition to tubulitis and interstitial nephritis, mesengiolysis, mesangial proliferation, mesangial edema, subendothelial thickening, endothelial thickening, lumen narrowing, fibrinoid necrosis of afferent arterioles, and microthrombi were observed. All of which are similar to patients with renal TA-TMA. Immunostaining showed C3 complement deposition in the glomeruli, and these glomerular lesions were attenuated in C3-deficient mice, suggesting that complement activation may also be involved in renal injury.

In the kidneys of mice following allo-HCT, increased expression of messenger RNAs for TNF-α, IFN-γ, IL-1α, IL-2, IL-6, and IL-10, as well as the adhesion molecules intercellular adhesion molecule 1 (ICAM-1) and vascular cell adhesion molecule 1 (VCAM-1) have been reported (106). Sadeghi et al. (114) performed BMT in mice using chemotherapy conditioning and compared kidney transcript expression patterns to those in the liver. Genes that were upregulated in the kidneys of allogeneic recipients, compared to syngeneic recipients and muscle without GVHD, included genes involved in antigen presentation, immune response, and leukocyte migration. These patterns were similar to those in the liver.

Collectively, these pre-clinical studies suggest that infiltration of donor-derived immune cells, changes in cytokines and chemokines, and activation of the complement system may be responsible for renal injury after allo-HCT.

## Prevention and Treatment of AKI After HCT

### Principles of Prevention and Treatment of AKI

AKI is triggered by a variety of factors related to HCT. Therefore, reducing these HCT-related complications is key to preventing AKI. One important strategy to reduce HCT-related complications is to tailor the choice of conditioning regimen and donor source according to each patient’s disease status and comorbidities. The largest contributor to AKI after HCT is drug-induced kidney injury, which can be mitigated by administrating appropriate doses of nephrotoxic agents, and using less nephrotoxic agents when possible. For example, limiting exposure to nephrotoxic antimicrobials decreases the incidence and severity of AKI (115).

Treatment of AKI depends on whether it is pre-renal, renal, or post-renal. Pre-renal AKI is caused by inadequate renal blood flow and responds to hydration. Hydration is also used to prevent renal injury from nephrotoxic agents such as IV contrast for imaging studies. However, care must be taken to avoid fluid overload and pulmonary edema in fluid-sensitive patients including those with decreased cardiac function. Renal AKI is unresponsive to hydration, and oliguria or anuria may persist for several weeks. Blood pressure and fluid balance should be tightly controlled, and nephrotoxic drugs should be discontinued. Depending on the cause of renal AKI, pharmacotherapy with furosemide, atrial natriuretic peptide, and low-dose dopamine may be used, but there is a lack of evidence for their efficacy in preventing or treating renal AKI. In the case of post-renal AKI, obstruction and hydronephrosis are diagnosed by imaging studies, and the main treatment is relief of the obstruction (12, 116).

Renal replacement therapy (RRT) is required for severe renal dysfunction. Patients with prolonged oliguria or anuria, for which RRT is essential for life support, are absolute indications for RRT. There is no consensus on whether earlier initiation of RRT improves the prognosis of severe AKI (117–120).

### Disease-Specific Treatment

When the cause of AKI is determined, treatment should focus on correcting it. For example, AKI related to hepatic SOS should be treated with defibrotide, aggressive attempts to maintain fluid balance, and possibly methylprednisolone (121, 122). Prophylactic use of ursodeoxycholic acid (123), defibrotide (124), and fresh frozen plasma (125) should be considered in those at high risk of SOS.

There is no established treatment for TA-TMA, but potential contributing factors should be eliminated when possible. For example, if an infection is thought to contribute, then treatment should be directed toward the pathogen, and every effort should be made to avoid further kidney injury. If CNI therapy is thought to contribute, then CNI withdrawal or dose reduction should be considered (62). Plasma exchange may be performed for the treatment of severe TA-TMA, but the response is usually poor (126, 127). Other potentially efficacious treatments include recombinant thrombomodulin (128), defibrotide (129), rituximab (a monoclonal antibody against CD20) (130), and eculizumab (a monoclonal antibody against complement C5) (131). However, none of these have been investigated in large-scale prospective studies.

Hemorrhagic cystitis (caused by adenovirus or BK virus infection) may require surgical decompression with a nephrostomy tube if urinary tract obstruction cannot be relieved by bladder irrigation from a urinary catheter. The antiviral drug cidofovir is effective for hemorrhagic cystitis caused by adenovirus (132, 133) and may be effective for hemorrhagic cystitis caused by BK virus (134). Ganciclovir (135) and valganciclovir (136) have also been reported to be effective against hemorrhagic cystitis caused by adenovirus.

## Conclusion

AKI is a common complication of HCT and an important determinant of HCT-related mortality. As described above, AKI after HCT can be caused by a variety of HCT complications and by many drugs commonly used before, during, and after HCT. Furthermore, the agents used to prevent and treat many HCT-related complications can contribute to kidney injury. In individual patients, it is common for several etiologies of AKI to be present at once. In fact, it is likely that these multiple etiologies act in combination. Due to the presence of multiple etiologies for AKI, it is often difficult to quantify the contribution of any one factor in individual patients. In addition, uncharacterized factors may also contribute to renal injury after HCT. For instance, the kidneys are not considered a primary aGVHD target organ, but recent data suggests that renal aGVHD may cause AKI.

Additional research is needed to identify the factors that cause AKI in HCT recipients. This research will hopefully improve the clinical ability to pinpoint specific causes of AKI in individual patients, and lead to therapies targeting each underlying pathologic etiology. Such advances in the diagnosis, prevention, and treatment of AKI in HCT recipients will improve the safety of HCT.

## Author Contributions

MM, KI, and TT conceived of the concept and important topics to include in the article. All authors contributed to writing this review and critical appraisal and review of the final version.

## Funding

This work was supported by JSPS KAKENHI Grant-in-Aid for Scientific Research (C) Grant Number JP20K08704 (TT), JP21K08410 (KI), The Japanese Society of Hematology Research Grant (TT), Takeda Science Foundation Research Grant (TT), The Hope from Harper St. Baldrick’s Foundation Fellowship (DP), and Hyundai Hope on Wheels Young Investigator Grant (DP).

## Conflict of Interest

The authors declare that the research was conducted in the absence of any commercial or financial relationships that could be construed as a potential conflict of interest.

## Publisher’s Note

All claims expressed in this article are solely those of the authors and do not necessarily represent those of their affiliated organizations, or those of the publisher, the editors and the reviewers. Any product that may be evaluated in this article, or claim that may be made by its manufacturer, is not guaranteed or endorsed by the publisher.
